# Screening of potentially active compounds against rheumatoid arthritis in the Juan-Bi decoction using systems pharmacology and animal experiments

**DOI:** 10.3389/fcell.2024.1396890

**Published:** 2024-06-25

**Authors:** Dahai Liu, Qiang Fu, Leyna G. Liu, Wenwen Li, Fei Qi, Justin Liu, Lifeng Shang, Xiu Wang, Fang Yang, Jie Li, Daoqiang Lu, Huiying Feng, Ziwen Zhang, Yiqing Chen, Junru Liang, Jiayi Yao, Hua Lv, Riwang Li, Jun Wang, Di Wu, Yuxi Liu, Chenglai Xia, Wenxing Li

**Affiliations:** ^1^ School of Medicine, Foshan University, Foshan, Guangdong, China; ^2^ College of Letters and Science, University of California, Berkeley, Berkeley, CA, United States; ^3^ Reproductive Medicine Center, Department of Obstetrics and Gynecology, The First Affiliated Hospital of Anhui Medical University, Hefei, Anhui, China; ^4^ School of Clinical Medicine, Anhui Medical College, Hefei, Anhui, China; ^5^ Department of Statistics, University of California, Riverside, Riverside, CA, United States; ^6^ Guangdong Andao Medical Apparatus and Instruments Co., Ltd, Foshan, Guangdong, China; ^7^ Department of Traditional Chinese Medicine and Gynecology, Shunde Women and Children’s Hospital of Guangdong Medical University (Maternity and Child Healthcare Hospital of Shunde Foshan), Foshan, Guangdong, China; ^8^ Foshan Maternity and Child Healthcare Hospital, Foshan, China; ^9^ Department of Biochemistry and Molecular Biology, School of Basic Medical Sciences, Southern Medical University, Guangzhou, Guangdong, China

**Keywords:** rheumatoid arthritis, traditional Chinese medicine, Juan-Bi decoction, systems pharmacology, bornyl acetate

## Abstract

**Background:** The Juan-Bi decoction (JBD) is a classic traditional Chinese medicines (TCMs) prescription for the treatment of rheumatoid arthritis (RA). However, the active compounds of the JBD in RA treatment remain unclear.

**Aim:** The aim of this study is to screen effective compounds in the JBD for RA treatment using systems pharmacology and experimental approaches.

**Method:** Botanical drugs and compounds in the JBD were acquired from multiple public TCM databases. All compounds were initially screened using absorption, distribution, metabolism, excretion, and toxicity (ADMET) and physicochemical properties, and then a target prediction was performed. RA pathological genes were acquired from the DisGeNet database. Potential active compounds were screened by constructing a compound–target–pathogenic gene (C-T-P) network and calculating the cumulative interaction intensity of the compounds on pathogenic genes. The effectiveness of the compounds was verified using lipopolysaccharide (LPS)-induced RAW.264.7 cells and collagen-induced arthritis (CIA) mouse models.

**Results:** We screened 15 potentially active compounds in the JBD for RA treatment. These compounds primarily act on multiple metabolic pathways, immune pathways, and signaling transduction pathways. Furthermore, *in vivo* and *in vitro* experiments showed that bornyl acetate (BAC) alleviated joint damage, and inflammatory cells infiltrated and facilitated a smooth cartilage surface via the suppression of the steroid hormone biosynthesis.

**Conclusion:** We screened potential compounds in the JBD for the treatment of RA using systems pharmacology approaches. In particular, BAC had an anti-rheumatic effect, and future studies are required to elucidate the underlying mechanisms.

## 1 Introduction

Rheumatoid arthritis (RA) is a systemic polyarticular chronic autoimmune joint disease that affects 0.5%–1.0% of the global population (Huang et al., 2021). RA primarily affects the musculoskeletal system, with joints commonly experiencing severe ramifications, including local inflammation, cartilage deterioration, and bone erosion. These cascading effects ultimately lead to joint damage and physical disability (Mueller et al., 2021). There are multiple immune function-related pathways correlated with RA that include the JAK/STAT signaling pathway, the NF-κB signaling pathway, the AMPK signaling pathway, and the TLR signaling pathway (Li et al., 2019a; Cheng et al., 2020; Li et al., 2020). Contemporary RA treatment for encompass several approaches, such as utilizing composite markers for gauging disease activity, implementing targeted therapy strategies, and employing a range of conventional, biological, and innovative non-biological anti-rheumatic drugs for managing the condition (Smolen et al., 2016). The majority of patients’ experience alleviation of associated symptoms following treatment; however, there remain individuals who do not respond to the existing therapy. Hence, there is an urgent need for novel treatment approaches.

There are limited pharmaceutical options available for the clinical treatment of RA. Nevertheless, significant progress has been made due to ongoing advancements in disease-modifying anti-rheumatic drugs (DMARDs) that have resulted in notable success in preventing and mitigating disease activity in RA patients. However, despite these advances, response rates remain constrained, underscoring the need for innovative targets and therapies (Huang et al., 2021). In recent years, there has been a growing emphasis on the role of traditional Chinese medicines (TCMs) and their extracts in disease treatment (Tang et al., 2021). These medicines include the Shuanghuanglian injection, the Danshen injection, the Tacrolimus injection, and other preparations that are effective compounds extracted and separated from Coptis Chinensis, Salvia miltiorrhiza, Panax ginseng, and Ophiopogon japonicus (He and Li, 2021). Whether it is a compound preparation with undisclosed ingredients or a small molecule drug with well-defined active components, both have supplied compelling evidence-based support for the utilization of TCM for the clinical transformation of RA treatment. This evidence indicates that the integration of TCM into a comprehensive treatment approach for RA patients can contribute significantly to achieving disease remission.

The Juan-Bi decoction (JBD), initially documented in the “Yang’s Household Recipe” authored by Yang Kui during the Southern Song Dynasty, primarily addresses symptoms such as “rheumatic discomfort, body aches, neck and arm pain, mobility challenges, cold extremities, and lumbar heaviness with weakened legs, muscles, and veins.” This prescription stands as a classical clinical remedy for the management of RA (LI et al., 2021). An investigation involving a combination of the Juan-Bi Tongluo decoction with methotrexate tablets and enteric-coated sulfasalazine tablets has demonstrated that this treatment effectively alleviates the primary clinical symptoms in patients with early RA, particularly those experiencing damp-heat arthralgia. Furthermore, it contributes to improvements in various laboratory physiological and chemical markers. These beneficial effects may be attributed to a reduction in adipokine levels and the regulation of the inflammatory immune response (Xiao and Ye, 2021). The outcomes from the combined approach of TCM and western anti-rheumatism medication for RA have indicated superior therapeutic efficacy, particularly in active patients. This improvement could potentially be linked to the amelioration of the erythrocyte sedimentation rate (ESR), rheumatoid factor (RF), and C-reactive protein (CRP) levels, as well as the regulation of MMP-3 and TIMP-1 levels in both serum and joint fluid (Bian, 2018). Furthermore, the combination of Qing-Re JBD with methotrexate has demonstrated a notable therapeutic impact in addressing damp-heat obstruction type RA. This treatment approach effectively ameliorates the clinical symptoms in patients, decreases the levels of serum α1-AGP and TL1A, and mitigates the inflammatory response, all without an increase in adverse reactions (Wang, 2021).

Previous pharmacological investigations, both *in vitro* and *in vivo* have indicated that the JBD exhibits promising protective and therapeutic properties. Moreover, the Qing-Re JBD has displayed significant clinical improvements in patients with RA characterized by cold-dampness obstruction. This treatment effectively reduces the ESR and CRP levels. The underlying therapeutic mechanism appears to involve the suppression of pro-inflammatory cytokines TNF-α and IL-1β, a decrease in the MMP-3 levels, and an upregulation of TIMP-1 expression (Li et al., 2019b). Rat experiments have revealed that the Cheng-Shi JBD exerts its anti-inflammatory properties by elevating serum IL-35 levels and suppresses the release of IL-17 (Lei et al., 2019). In addition, the JBD additionally modulates immune balance by diminishing natural killer cells and fostering T-regulatory cells in rats with collagen-induced arthritis (CIA). Furthermore, it lowers the cAMP levels in T lymphocytes in rats with adjuvant-induced arthritis (Wang et al., 2020).

The JBD is a renowned TCM formulation utilized for RA treatment. However, our understanding of its effectiveness primarily relies on TCM knowledge. There has been limited research on the active components and potential mechanisms of action of the JBD and its constituent herbs. The integration of network pharmacology and systems pharmacology offers a promising avenue for precise and effective therapeutic interventions. This approach leverages synergistic multi-compound networks and the repurposing of drugs, bypassing the need for extensive drug discovery and expediting clinical translation. The use of robust and meticulous methodologies can help researchers analyze potential synergies and facilitate more comprehensive assessments for future applications (Panossian et al., 2021; Nogales et al., 2022). In this study, we used a systematic approach that involves constructing a compound-target-pathogenic gene network using the chemical constituents found in the herbal components of the JBD. We used a mathematical model to optimize this network and successfully identified and screened the active ingredients within these herbal components. Subsequently, we validated these findings through experimentation. This comprehensive methodology presents a promising avenue for utilizing the JBD in RA treatment. It not only furnishes a theoretical foundation but also serves as a valuable methodological reference for the screening of natural products in TCM.

## 2 Methods

### 2.1 Herbs and compounds of the JBD

Botanical drugs and dosage details in the JBD study were sourced from the Chinese Pharmacopeia 2020 (National Pharmacopoeia Commission, 2020). Taxonomic validation of all botanical drugs was conducted using the Plants of the World Online (https://powo.science.kew.org/) and the Chinese Medicinal Material Images Database (https://library.hkbu.edu.hk/electronic/libdbs/mmd/) (Table 1). Information about the chemical components within these botanical drugs was gathered from various publicly accessible Traditional Chinese Medicines (TCMs) databases, such as the Traditional Chinese Medicine Information Database (TCMID, http://bidd.group/TCMID/) (Chen et al., 2006), and the Traditional Chinese Medicine and Active Ingredient Database (TCMAID, http://www.organchem.csdb.cn/scdb/main/tcm_introduce.asp). The chemical structures were prepared and converted into canonical SMILES using the OpenBabel Toolkit (O Boyle et al., 2008).

**TABLE 1:** Herbs and dosage information in JBD.

Herb Chinese name	Herb Latin name	Dose (g)
Bai Shao	*Paeonia lactiflora* Pall. (Paeoniaceae, Paeoniae Radix Alba)	9
Dang Gui	*Angelica sinensis* (Oliv.) Diels (Apiaceae, Angelicae Sinensis Radix)	9
Fang Feng	*Saposhnikovia divaricata* (Turcz.) Schischk. (Apiaceae, Saposhnikoviae Radix)	9
Gan Cao	*Glycyrrhiza uralensis* Fisch. (Fabaceae, Glycyrrhizae Radix et Rhizoma)	3
Huang Qi	Astragalus membranaceus (Fisch.) Bge. (Fabaceae, Astragali Radix)	9
Jiang Huang	Curcuma longa L. (Zingiberaceae, Curcumae Longae Rhizoma)	9
Qiang Huo	Notopterygium incisum Ting ex H. T. Chang (Apiaceae, Notopterygii Rhizoma et Radix)	9
Sheng Jiang	*Zingiber officinale* (Willd.) Rosc. (Zingiberaceae, Zingiberis Rhizoma Recens)	3

### 2.2 Screening of potentially active compounds in the JBD

The absorption, distribution, metabolism, excretion, and toxicity (ADMET) properties and physicochemical characteristics of all compounds were predicted using the ADMETlab 2.0 web server (https://admetmesh.scbdd.com/) (Xiong et al., 2021). Under Lipinski’s rule of five (Pollastri, 2010), potential active compounds were required to fulfill the following criteria: (1) molecular weight (MW) ≤ 500; (2) number of hydrogen bond donors (nHD) ≤ 5; (3) number of hydrogen bond acceptors (nHA) ≤ 10; (4) logarithm of the n-octanol/water distribution coefficient (logP) ≤ 5; and (5) the number of rotatable bonds (nRot) ≤ 10. Furthermore, potential active compounds needed to meet the following additional conditions: (6) human oral bioavailability (OB) ≥ 30%, (7) Caco-2 permeability (Caco-2) ≥ 0.4, (8) topological polar surface area (TPSA) ≤ 60, and (9) no hepatotoxicity.

### 2.3 Compound-target prediction

The potential targets of the potential active compounds were predicted using the following web servers: the similarity ensemble approach (SEA) search server (https://sea.bkslab.org/) (Keiser et al., 2007), HitPickV2 (http://www.hitpickv2.com/) (Hamad et al., 2019), and SwissTargetPrediction (http://www.swisstargetprediction.ch/index.php) (Daina et al., 2019). The union set of the prediction results was defined as the potential targets.

### 2.4 Collection of RA pathological genes

The RA pathological genes were searched and downloaded from the DisGeNet database (https://www.disgenet.org/) (Pinero et al., 2020). Genes with literature reports were defined as RA pathological genes. The intersection of targets and pathogenic genes was defined as essential common proteins (ECPs).

### 2.5 Compound-target-pathological gene (C-T-P) network construction

For the network construction methods, see our previous publication (Xu et al., 2023a). Briefly, we retrieved the interactions between targets and pathogenic genes and extracted documented protein-protein interactions from the BioGrid database (https://thebiogrid.org/) (Oughtred et al., 2019). We combined this with previous compound-target prediction results, and the compound-target-pathological gene (C-T-P) network was constructed and visualized using Cytoscape software (Kohl et al., 2011).

### 2.6 RA transcriptome data analysis

Transcriptome data from the macrophages of RA patients were downloaded from the National Center for Biotechnology-Gene Expression Omnibus (NCBI-GEO) database (https://www.ncbi.nlm.nih.gov/geo/) with accession identification numbers of GSE10500 and GSE97779. Data preprocessing, integration, and global normalization were conducted using our previously described method (Li et al., 2016a; Li et al., 2016b). The differential expression analysis was conducted using the empirical Bayesian algorithm in the limma package (Ritchie et al., 2015) in R statistical software (https://www.r-project.org/). Significantly differentially expressed genes were defined as absolute values of the log2-transformed fold change (logFC) > 1 and an FDR *p*-value <0.05.

### 2.7 Optimization of the C-T-P network using the multi-objective optimization (MOO) model

The C-T-P network was optimized using the MOO model. The purpose of optimization was to extract target genes that were strongly associated with compounds and pathogenic genes and were dysregulated in RA patients. Details of the MOO model and network optimization can be found in a previous report (Xu et al., 2023b).

### 2.8 Gene Ontology (GO) and the Kyoto Encyclopedia of Genes and Genomes (KEGG) enrichment analysis

The GO terms of biological process, cellular component, molecular function, and human gene information were downloaded from the QuickGO database (https://www.ebi.ac.uk/QuickGO/) (Binns et al., 2009). The reference human genes and pathways were obtained from the Kyoto Encyclopedia of Genes and Genomes (KEGG) database (http://www.kegg.jp/) (Kanehisa and Goto, 2000). The GO terms and KEGG pathways with fewer than 10 genes were removed. The enrichment analysis was performed using the hypergeometric test. An false discovery rate (FDR)-corrected *p*-value ≤0.05 was considered significantly enriched.

### 2.9 Comparison of the MOO method with other models

The degree model, closeness model, and betweenness model (Yang et al., 2021) were used to optimize the constructed C-T-P network and compared with the MOO model. For details see the previous publication (Xu et al., 2023a). The optimization performance of the different models was compared from the five aspects: (1) the coverage of essential common proteins; (2) the coverage of the top 100 enriched KEGG pathways; (3) the coverage of the top 1,000 enriched GO biological processes (BP); (4) the average regulating intensity; and (5) the cumulative differential expression. Genes in the optimized network in the different models were used to perform the KEGG pathway and GOBP enrichment analyses, and the reference KEGG pathways and Gene Ontology biological processes (GOBPs) were enriched using essential common proteins (ECPs). The average regulating intensity was calculated as the mean Pearson’s correlation coefficient for the gene-gene pairings in the optimized network. The cumulative differential expression was defined as the sum of the absolute values of the logFC of ECPs in the optimized network.

### 2.10 Calculation of the interaction intensity flow on the pathogenic genes

We extracted the pathogenic genes correlated with each compound in the optimized network and then calculated the interaction intensity flow of each pathogenic gene. Let be the interaction intensity flow of the pathogenic gene; is the set of target genes that interact with the pathogenic gene; and is the number of target genes in. The following equation was used for the calculation:

$${Intensity}_{k}=\left\{\begin{array}{l} S_{{MOO}_{k}}^{\prime },k\in T,k\in P\;\\ \frac{\sum S_{{MOO}_{j}}^{\prime }}{n_{k}},k\notin T,k\in P,j\in T_{k} \end{array}\right.$$

Where is the interaction intensity of compound, on pathogenic genes; and is the set of pathogenic genes that correlate with compound. The equation is:

$${Intensity}_{i}=\sum_{k=1}^{C_{k}}{Intensity}_{k},k\in C_{k}$$

The above formula was used to calculate the interaction intensity flow of a single compound on the pathogenic genes. For the interaction intensity of multiple compounds on the pathogenic genes, we first constructed a list of combinations of the different compounds and then calculated the interaction intensity of each combination using the above equation. The combination containing the same number of compounds with the strongest interaction intensity was defined as the optimal combination. Finally, we sorted these optimal combinations in ascending order of the interaction intensities and selected the least compound combination whose cumulative interaction intensity reached 90% of the interaction intensity of all the compounds in the optimized network.

### 2.11 Calculation the cumulative interaction intensity flow on the pathway

The cumulative interaction intensity flow of the pathway is defined as the sum of the interaction intensity flow of pathogenic genes in the pathway. Let be the cumulative interaction intensity flow of pathway; is the number of pathogenic genes in pathway; as the number of pathways that a pathogenic gene is involved in. The calculation equation is as follows:

$${Intensity}_{p}=\sum_{k=1}^{m}\frac{{Intensity}_{k}}{n_{k_{p}}}$$

All pathways are sorted according to the, and the pathways with larger values are considered to be the primary pathways affected by the JBD.

### 2.12 Cell line and cell culture

The RAW264.7 cell line was shared by the South China University of Technology. They were cultured in the RAW264.7 cell medium, containing Dulbecco’s Modified Eagle Medium (DMEM), 10% fetal bovine serum (FBS), and a 1% penicillin-streptomycin (P/S) solution at 37°C and 5% CO2 (Procell, Wuhan, China). A total of 1 μg/ml of lipopolysaccharide (LPS) (Biosharp, Hefei, China) was administered to the RAW264.7 cells for 24 h to construct an inflammatory cell model.

### 2.13 CCK8 assay

The RAW264.7 cells were cultured in 96-well plates and treated with 100, 50, 25, 12.5, 6.25, 3.125, and 1.56 μM compounds for 24 h. A total of 10 μL of the CCK8 reagent (Jiancheng Bioengineering Institute, Nanjing, China) was added to the RAW264.7 cells at 37°C for 1.5 h. The absorbance was analyzed at 450 nm using a microplate reader (Bio-Rad, Hercules, CA, USA) with wells without cell serving as blanks. The cell proliferation was expressed by the absorbance.

### 2.14 Nitric oxide assay

The RAW264.7 cells were cultured in 48 well plates and incubated for 24 h to measure the anti-inflammatory activity by nitric oxide (NO) assay. The cells were treated with 25, 10, 5, 1, and 0 μM compounds at 37°C for 24 h. Subsequently, LPS (0 or 1 μg/mL) was administered into the cells and then incubated for 24 h. NO production in the supernatant was measured using the nitrite measurement according to the manufacturer’s protocol. In brief, an equal volume of supernatant and Griess reagent were mixed and incubated for 10 min at ambient temperature in the dark, and the absorbance was measured at 570 nm using a Synergy HT microplate reader from Bio-Tek Instruments Inc. (Winooski, VT, USA).

### 2.15 Ethics statement

This study was conducted in strict accordance with the recommendations of the Guide for the Care and Use of Laboratory Animals of the National Institutes of Health. All methods were conducted in accordance with the Ethics Committee Institute of the School of Medicine of Foshan University. All experimental protocols were approved by Foshan University. All methods are reported in accordance with the Animal Research: Reporting of *In Vivo* Experiments (ARRIVE) guidelines. All surgeries were performed under chloroform anesthesia, and all efforts were made to minimize suffering.

### 2.16 Construction of the collagen-induced arthritis mouse model

The exact number of experimental units allocated to each group was six mice. The total number in each experiment was 30 mice. The total number of animals used was 90 mice. DBA/1J mice (males, 8 weeks old, free of murine-specific pathogens) were obtained from the Experimental Animal Center, Xiangya School of Medicine, Central South University. Random numbers were generated using the standard = RAND () function in Microsoft Excel. Randomization was conducted as follows. Animals were assigned a group designation and weighed. A total number of 30 animals were divided into five different weight groups (six animals per group). Each animal was assigned a temporary random number within the weight range group. Cages were given a numerical designation based on their position on the rack. For each group, a cage was selected randomly from the pool of all cages. One or two animals were removed from each weight range group and given their permanent numerical designation in the cages.

Mice were kept under illumination conditions, with the room temperature maintained at 22°C ± 4°C, and they were granted free access to food and water. The mice were adaptively fed for 7 days, and then procedures were conducted in the animal room of Foshan University. Each mouse was injected with 100 μL bovine type II collagen (2 mg/mL in 0.05 M acetic acid; Chondrex, Inc., Redmond, WA, USA) emulsified in complete Freund’s adjuvant (Sigma-Aldrich, St. Louis, MO, USA) on the day 7. On day 28, mice were administered 100 μL bovine type II collagen emulsified in incomplete Freund’s adjuvant by enhanced intradermal injection. Beginning on day 28, mice were administered methotrexate (MTX) (1 mg/kg) or JBD (20 mg/kg or 40 mg/kg) every 3 days. Complete adjuvants produce antibodies in mice, including IgG2a antibodies, which play an important role in the activation of the complement and subsequent development of arthritis, depending on the amount of tuberculosis bacteria in the complete adjuvant. Type II collagen and full adjuvant emulsification significantly induced widespread joint inflammation in CIA-susceptible DBA/1 mice. Mice were randomly divided into five groups, including control, CIA, CIA + MTX, CIA + BAC-L, and CIA + BAC-H. The mice were anesthetized with isoflurane and euthanized by means of cervical dislocation on day 40. Blood, knee, elbow, wrist, and ankle joints were collected from the mice. The following parameters were assessed: bone surface/volume ratio, H&E staining, safranin O-fast green staining, Western blot, and RT-qPCR.

The arthritis index (AI) was graded as following criteria: 0, normal joints; 1, slight swelling or a digital red spot; 2, red skin and slight swelling in the ankles and feet; 3, moderate swelling and erythema; 4, severe swelling and erythema involving the entire posterior claw or precipitation (Kollias et al., 2011).

### 2.17 Analysis of micro-CT

A Sample of mice knee joints was selected for micro-computed tomography (CT) scanning (ZKKS-MCT-Sharp, Guangzhou, China). The voltage was 70KV, the power was 7 W, four superimposed frames were used, the angle gain was 0.72°, the exposure time was 100 ms, and the rotation was 1 week to complete the scanning for the micro-CT. A three-dimensional image screenshot and bone parameter analysis were performed after scanning and reconstruction.

### 2.18 H&E staining and safranin O/fast green staining

The mice were anesthetized and sacrificed. The wrist, knee, ankle, and elbow joints were collected and fixed with 4% paraformaldehyde for 24 h and decalcified with 10% ethylenediaminetetraacetic acid (EDTA). After processing using conventional histological procedures, such as dehydration, transparency, wax immersion, and embedding, the paraffin-embedded tissue sections were stained by hematoxylin and eosin (H&E) staining (Sigma). The H&E staining score evaluation criteria were as follows: it could be divided into five degrees of zero to four points according to the edema, structural damage, and inflammatory cell infiltration of lesions, from light to heavy. The score was measured (Liu et al., 2022).

For safranin O/fast green staining, the paraffin-embedded slices were deparaffinized, sequentially immersed in xylene I for 20 min, xylene II for 20 min, absolute ethanol I for 5 min, absolute ethanol II for 5 min, 75% alcohol for 5 min and washed with tap water. The slices were stained with fast green staining for 5–10 min, dried to discard the excess dyeing solution until the cartilage was colorless, soaked in a differentiation solution, and washed in tap water. The slices were stained with the safranin staining solution for 15–30 s and dehydrated quickly with absolute ethanol. The slices were made transparent with xylene for 5 min, mounted with neutral gum, observed, and photographed under the microscope (Zhang et al., 2021a).

### 2.19 Western blot

The RAW264.7 cells or synovial tissues were homogenized and lysed in radioimmunoprecipitation assay (RIPA) buffer (Sigma-Aldrich) that contained a protease inhibitor (Roche, Mannheim, Germany). A total of 50 μg protein isolated from the RAW264.7 cells or synovial tissues was separated using 10% sodium dodecyl sulfate-polyacrylamide gel electrophoresis (SDS-PAGE) and transferred to a polyvinylidene difluoride (PVDF) membrane (Millipore, Billerica, MA, USA). The membranes were incubated with the following primary antibodies: CYP17A1 (ab125022, Abcam), HSD17B3 (ab126228, Abcam), TLR4 (ab13556, Abcam), p-MAPK (AF4001, Affinity), MAPK (BF8015, Affinity), p-p65 (#11011, SAB) and p65 (#48676, SAB). Subsequently, a horseradish peroxidase (HRP)-conjugated secondary antibody was added for the 1-h incubation. The blots were developed using the ECL Western blot Kit (LabLead, E1050) on the gel imaging system (ChampgelTM 5,000 plus, SINSAGE, China). The exposure time for blots ranged from 100 ms to 3 s. Film density was measured using ImageJ densitometry software and was normalized against glyceraldehyde 3-phosphate dehydrogenase (GAPDH) (#60004-1-Ig, Proteintech) (Liu et al., 2022).

### 2.20 RT-qPCR

The total RNA was extracted using Trizol (Invitrogen) according to the manufacturer’s protocol. One milligram of RNA was reverse transcribed into cDNA using avian myeloblastosis virus (AMV) reverse transcriptase (Qiagen, Vento, Netherlands) with an RNase inhibitor and oligo d(T) primer at 40°C for 50 min followed by heating at 90°C for 5 min. Then, 1 μL of the reverse-transcript was added to a 30 μL PCR mixture for 40 cycles. Each cycle included 93°C for 30 s and 54°C for 60 s and used Taq polymerase. Negative controls consisted of an equal volume of water substituted for the volume of RNA in the reverse transcriptase (RT) reaction. The normalization of mRNA expression data for sample-to-sample variability in RNA input, RNA quality, and reverse transcription efficiency was achieved by a the comparison between the copy numbers of the target gene and the housekeeping gene, and GAPDH. The relative gene expression was measured using the 2^−ΔΔCT^ method. Primer sequences are shown in Table 2.

**TABLE 2:** Oligonucleotide primers for the RT-qPCR.

Mouse gene	Primer sequence
CYP17A1	Forward: 5′-GCA​TCA​TAG​ACA​ACC​TGA​GCA​A-3′
	Reverse: 5′-GGG​TTT​TGT​TGG​GGA​AAA​TC-3′
HSD17B3	Forward: 5′-TTG​TTT​GGG​CCG​CTA​GAA​G-3′
	Reverse: 5′-CAC​CCA​CAG​CGT​TCA​ATT​CA-3′
TLR4	Forward: 5′-GAA​AGC​CGC​CTC​TTT​CCT​TC-3′
	Reverse: 5′-ATC​TCG​GGG​CAG​ATC​CTT​GT-3′
p-MAPK	Forward: 5′-CAG​TGG​AAG​GAC​AGC​ACA​AT-3′
	Reverse: 5′-TGG​TAT​CGC​CTT​TGC​CCA​TT-3′
MAPK	Forward: 5′-AAG​CAA​TGA​GAC​GAT​GAG​GCT-3′
	Reverse: 5′-CCC​CAC​GGA​CAG​TTT​GAT​TCT-3′
GAPDH	Forward: 5′-TTG​CAG​CTC​CTT​CGT​TGC​C-3′
	Reverse: 5′-GACCCA TTCCCACCA TCACA-3′

### 2.21 Statistical analysis

All experiments were run in a minimum of triplicates, and PRISM (GraphPad, La Jolla, CA, USA) statistical software was used for the statistical analysis. Statistical differences between two groups were determined using a Mann-Whitney test. The Kruskal–Wallis test determined differences among multiple groups. The effects of different concentrations of bornyl acetate on the CIA mouse model were assessed using a Mann-Whitney test. The Kruskal–Wallis test determined differences among multiple groups. All data are presented as mean values ±standard deviation (SD). *p* < 0.05 was considered statistically significant.

## 3 Results

### 3.1 Original and filtered potential active compounds in the JBD

**FIGURE 1:**
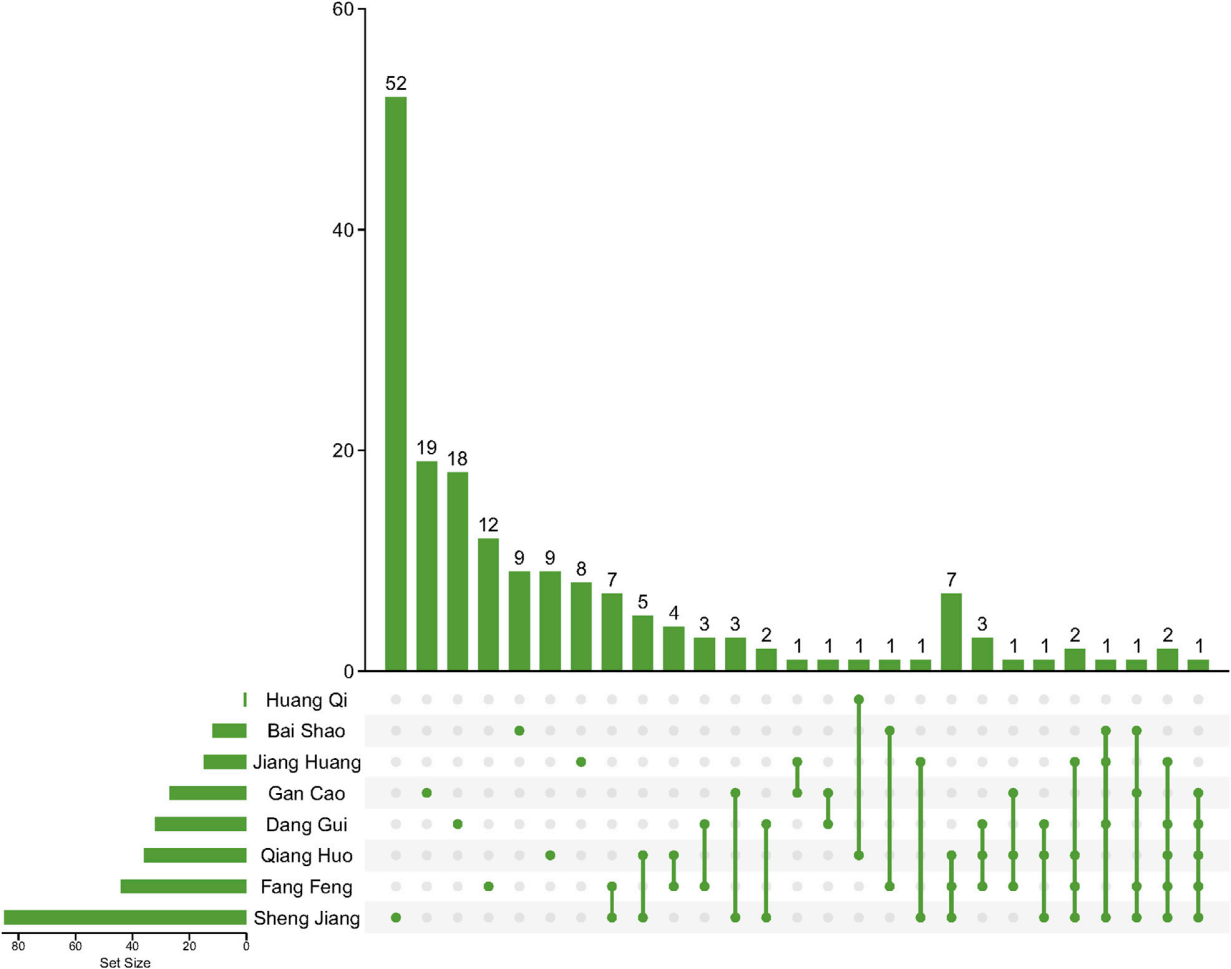
Compounds after ADMET screening in herbs in JBD. Lines between dots indicate herbs that share these compounds.

This study involved the collection of a total of 1,252 compounds derived from eight different herbs (Bai Shao, Dang Gui, Fang Feng, Gan Cao, Huang Qi, Jiang Huang, Qiang Huo, and Sheng Jiang). The pharmacological and ADMET properties of these compounds were assessed and predicted (Supplementary Table S1). Following our screening criteria, we identified and retained 274 potentially active compounds. Among these, the numbers of screened potential active compounds found in Bai Shao, Dang Gui, Fang Feng, Gan Cao, Huang Qi, Jiang Huang, Qiang Huo, and Sheng Jiang were 12, 33, 46, 27, 1, 16, 39, and 100, respectively (Figure 1; Supplementary Table S2). In addition, it is worth noting that there were 48 compounds that were common to two or more of these herbs.

### 3.2 C-T-P network construction and optimization

**FIGURE 2:**
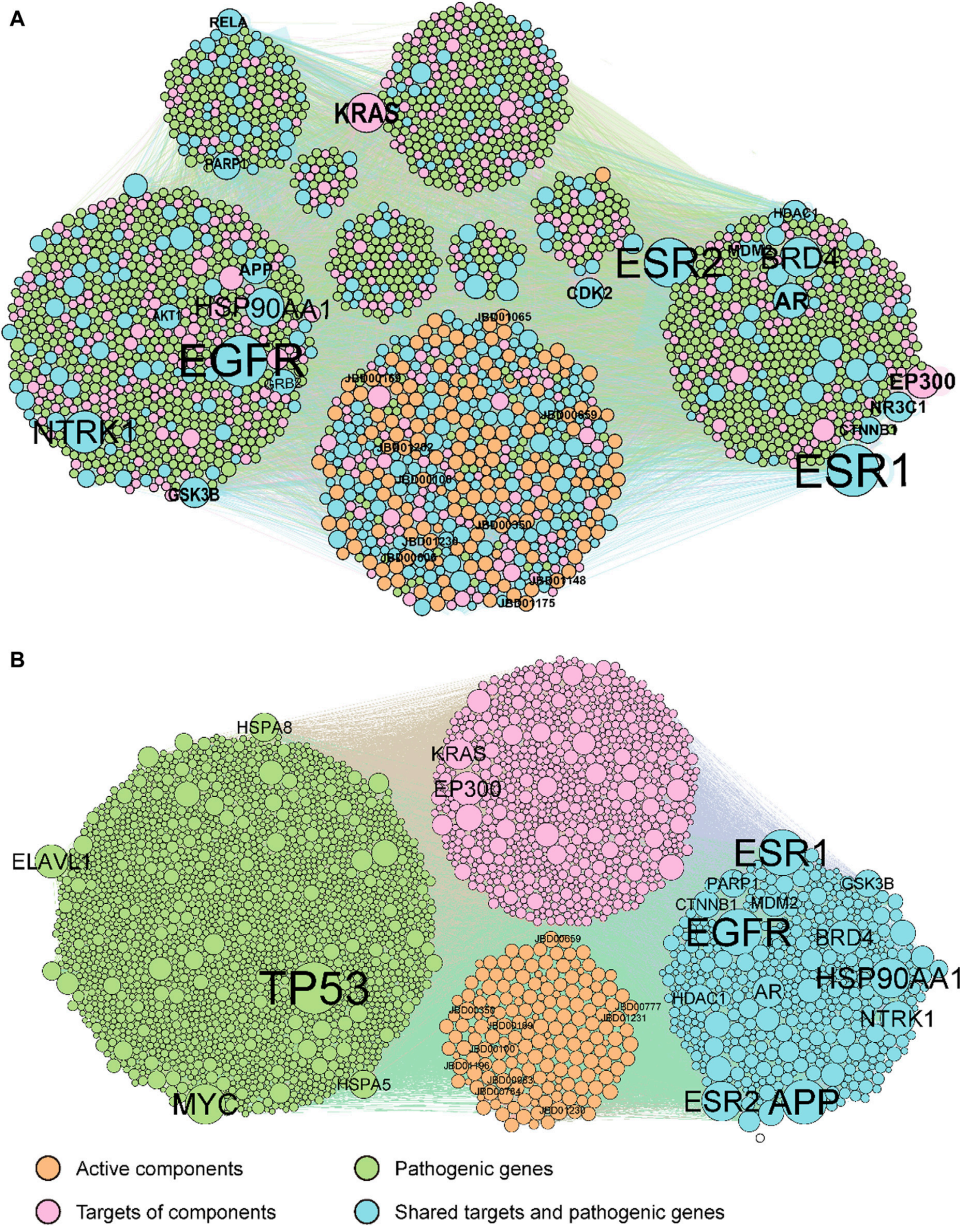
The original **(A)** and optimized **(B)** C-T-P network. The orange, pink, green, and cyan points indicate potential active compounds, predicted targets, RA pathogenic genes, and ECPs, respectively. Compounds and genes with a high degree of expression were highlighted and labelled.

Our methodology involved a series of steps. Initially, duplicate compounds were removed, after which target prediction was conducted using three different tools: SEA, HitPickV2, and SwissTargetPrediction, resulting in predictions of potential targets for the remaining 175 compounds. These predictions indicated that these compounds could potentially interact with a range of 16–178 targets (Supplementary Table S3). Furthermore, we collected a total of 2,766 distinct pathological genes associated with rheumatoid arthritis (Supplementary Table S4). These targets and pathological genes were cross-referenced with the BioGRID database to acquire protein-protein interaction data. Subsequently, we merged the compound-target network with the protein-protein interaction network to create the comprehensive C-T-P network. The original C-T-P network encompassed (Ru et al., 2014), 535 compound-target pairs and 46,838 protein-protein interactions (Figure 2A). Notably, 438 proteins were found to be both target proteins and pathogenic genes. Subsequent KEGG pathway and GOBP enrichment analyses highlighted their involvement in multiple signaling pathways linked to human diseases (Supplementary Tables S5, S6).

The original C-T-P network was refined using the MOO method. The optimized C-T-P network now comprises 10,239 compound-target pairs and 24,091 protein-protein interactions, incorporating 175 compounds, 1,085 predicted targets, and 2,081 rheumatoid arthritis pathological genes (with 438 genes shared between the target and pathological gene categories), as depicted in Figure 2B. Notably, the top 20 genes with the highest degrees in both the original and optimized networks have been labeled. Of these top genes, 13 are common to both the target genes and pathological genes (AR, APP, BRD4, CTNNB1, ESR1, ESR2, GSK3B, HDAC1, HSP90AA1, MDM2, NTRK1, PARP1, and NTRK1).

### 3.3 Comparison of the optimization performance between the MOO model and other models

In this study, we conducted a comprehensive examination of unique genes within the optimized network generated by the MOO model, as well as networks created using the degree model, closeness model, and betweenness model. These genes were subsequently subjected to overlap assessments concerning the essential genes, the KEGG pathway enrichment, and the GOBP enrichment. To gauge the model performance, we focused on the top 100 enriched KEGG pathways and the top 1,000 enriched GOBPs.

**FIGURE 3:**
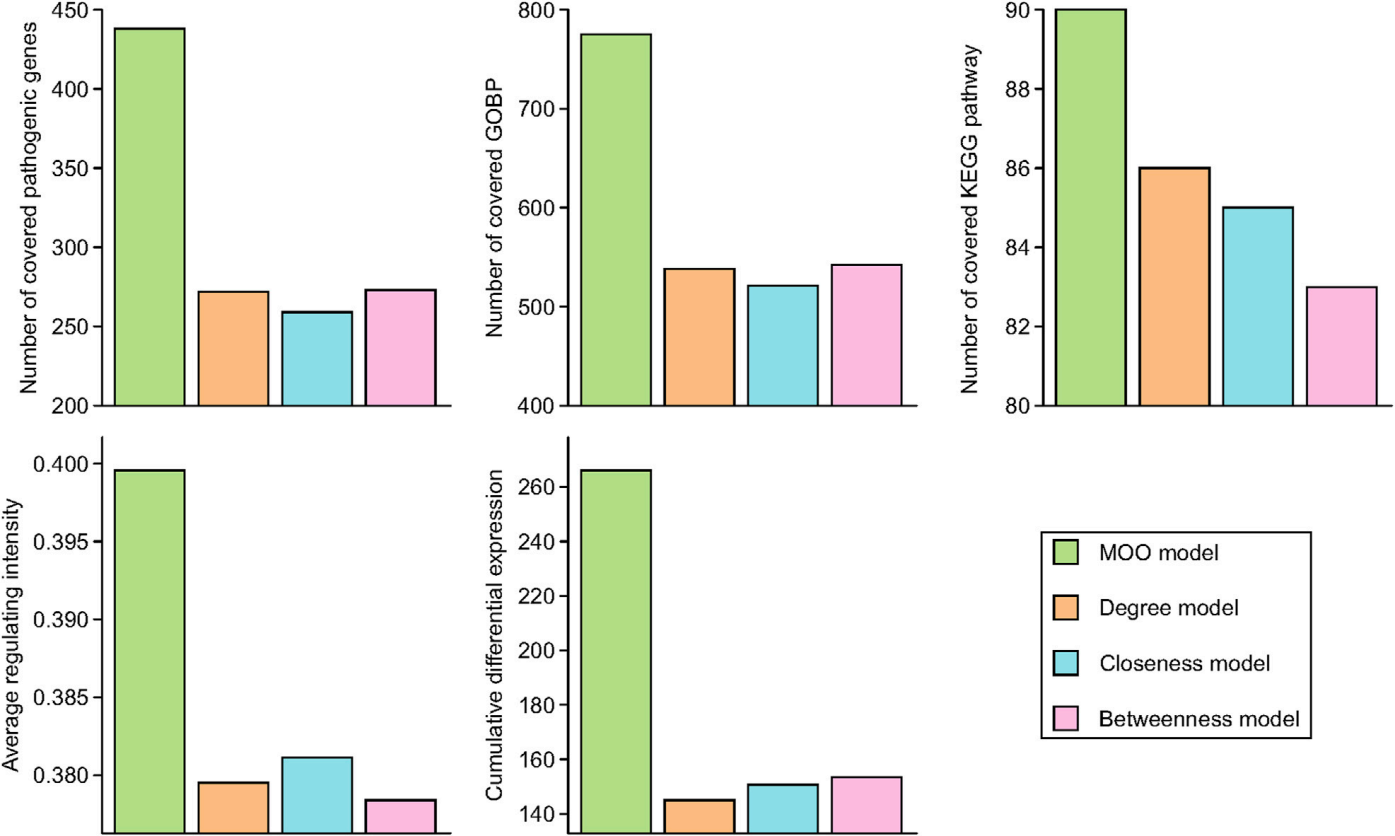
Optimization performance comparison of MOO model with other models. Essential common genes were defined as the overlapped target genes and RA pathogenic genes. Top 100 KEGG pathways and top 1000 GOBP based on FDR *p*-values were chose for comparison.

The results revealed varying degrees of overlap in the essential genes, with 438, 272, 259, and 273 common genes identified in the MOO model, degree model, closeness model, and betweenness model, respectively. Similarly, for the top 100 enriched KEGG pathways, there were 90, 86, 85, and 83 shared pathways among these four models, respectively. Furthermore, in the case of the top 1,000 enriched GOBPs, the numbers of overlapping terms were 775, 538, 521, and 542 in the MOO model, degree model, closeness model, and betweenness model, respectively. In addition, we evaluated the average regulating intensity and cumulative differential expression values, finding that these metrics were consistently higher in the MOO model compared with the other three models (Figure 3). These findings collectively indicated that the optimization performance of the MOO model surpassed that of the other models.

### 3.4 Active compound screening based on the interaction intensity

**FIGURE 4:**
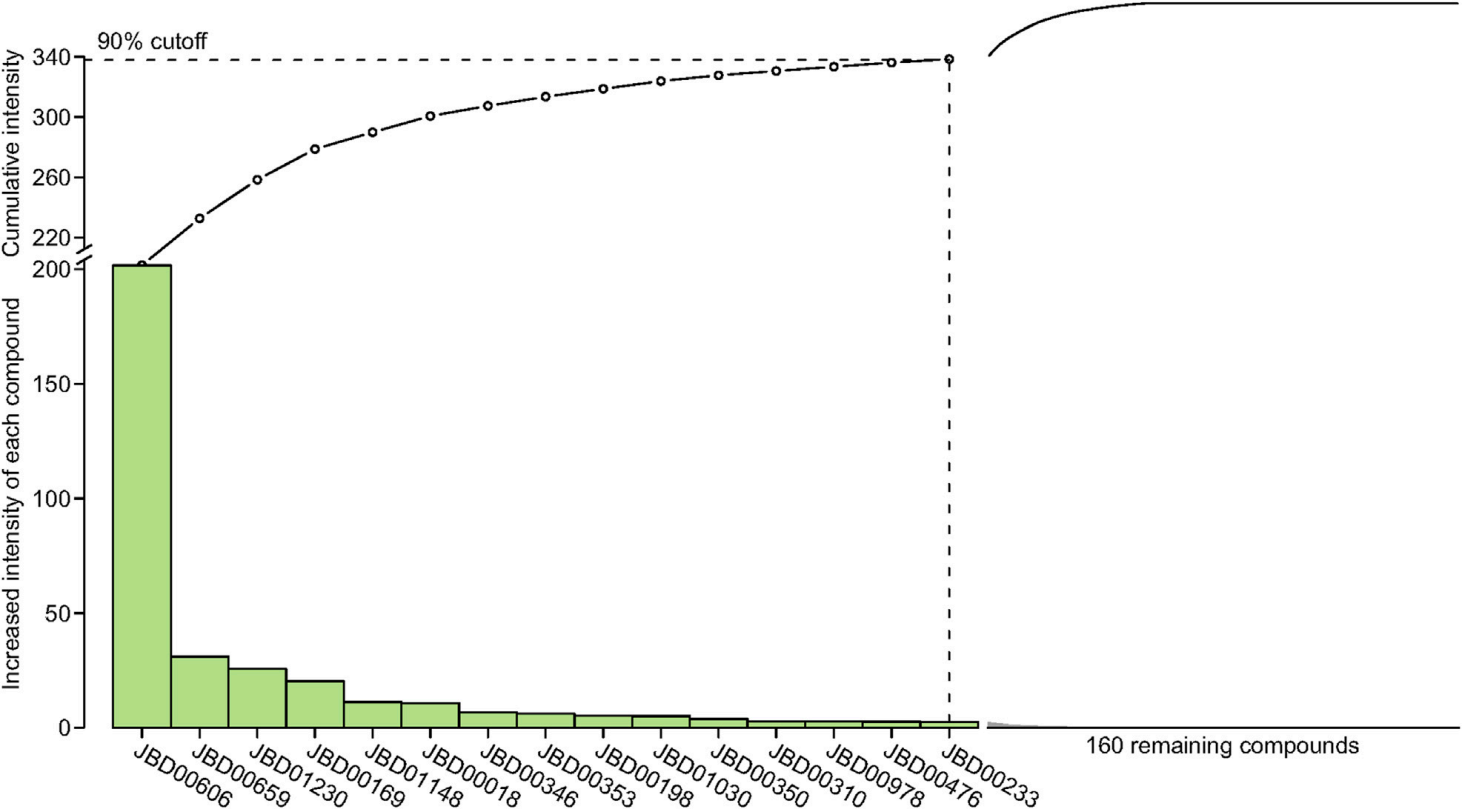
The interaction intensity on pathogenic genes of compounds in the optimized network. The cumulative intensity represents the combined interaction intensity on pathogenic genes of that compound and all existing active compounds. The increased intensity of each compound represents the cumulative intensity minus the interaction intensity on pathogenic genes of the existing active compounds. A dashed horizontal line indicates the 90% interaction intensity on pathogenic genes of all active compounds.

**FIGURE 5:**
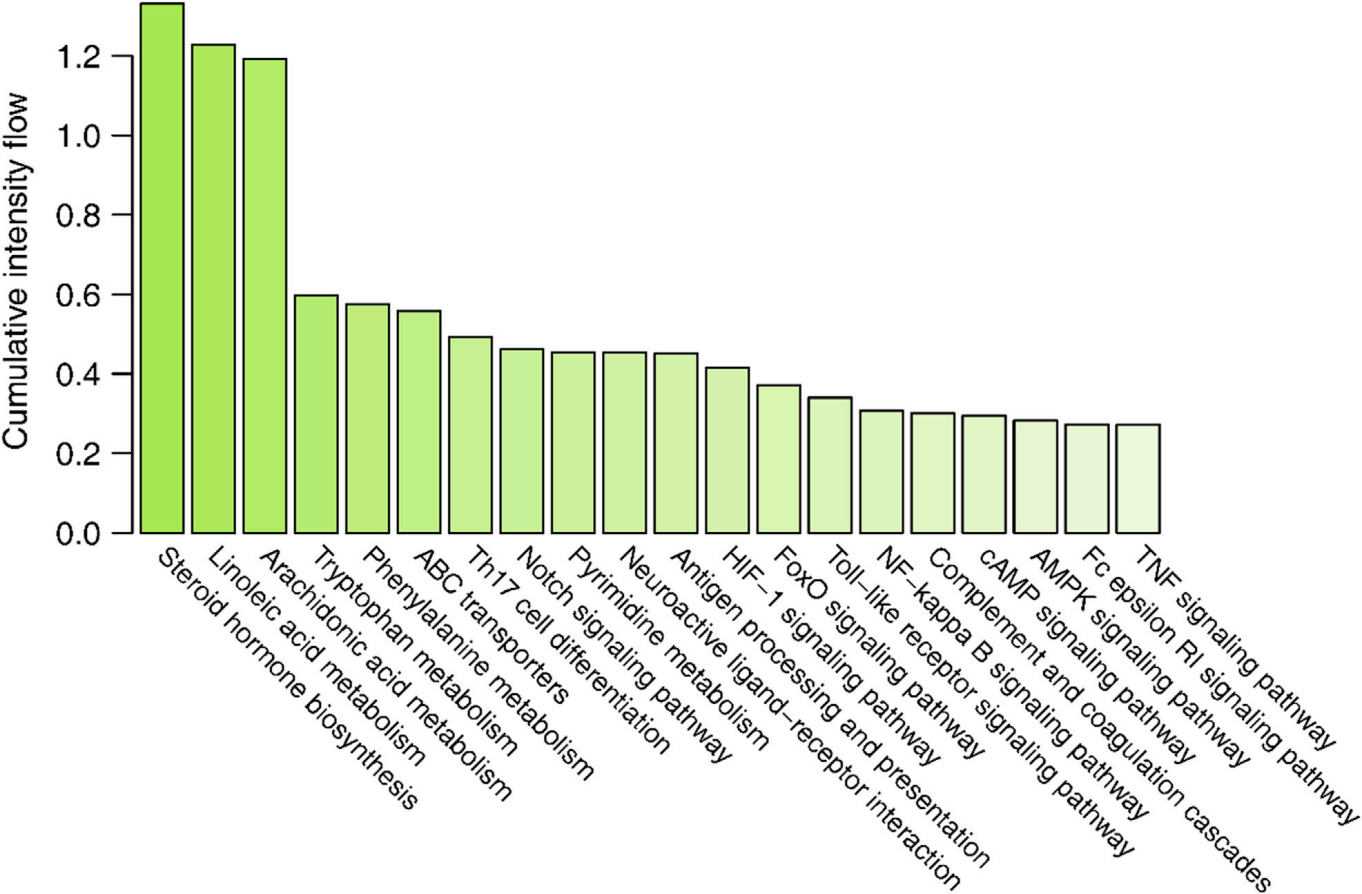
The cumulative interaction intensity flow of pathways. All pathways were sorted by descending cumulative intensity flow and the top 20 pathways were showed.

The interaction intensity flow on the pathogenic genes associated with each screened compound was calculated (Supplementary Table S7). Remarkably, the cumulative impact of 15 specific compounds on these pathogenic genes accounted for 90% of all active compounds (Figure 4). The details of these compounds, which originate from Bai Shao (one compound), Dang Gui (three compounds), Fang Feng (seven compounds), Gan Cao (four compounds), Huang Qi (one compound), Qiang Huo (three compounds), and Sheng Jiang (eight compounds), are elaborated in Table 3. Notably, among these compounds, JBD00018 (stearic acid) and JBD01030 (eugenol) were FDA-approved drugs, while the remaining 13 compounds are natural ingredients derived from botanical sources. The cumulative interaction intensity flow of these screened compounds on the enriched pathways suggested that these 15 compounds primarily affect metabolic pathways, immune pathways and signaling transduction pathways (Figure 5; Supplementary Table S8). Next, we verified the effectiveness of these compounds using cell and animal experiments.

**TABLE 3:** Final screened compound information in JBD.

ID	Name	Formula	Herbs
JBD00606	(Z)-1-(2,4-dihydroxyphenyl)-3-phenylprop-2-en-1-one	C_15_H_12_O_3_	Gan Cao
JBD00659	Xambioona	C_25_H_24_O_4_	Gan Cao
JBD01230	Dihydrocapsaicin	C_18_H_29_NO_3_	Sheng Jiang
JBD00169	Sedanolide	C_12_H_18_O_2_	Dang Gui, Fang Feng
JBD01148	6-shogaol	C_17_H_24_O_3_	Sheng Jiang
JBD00018	Stearic acid	C_18_H_36_O_2_	Bai Shao, Dang Gui, Fang Feng, Gan Cao, Huang Qi, Qiang Huo, Sheng Jiang
JBD00346	Lanceol	C_15_H_24_O	Fang Feng, Sheng Jiang
JBD00353	Heptadeca-1,8-dien-4,6-diyn-3,10-diol	C_17_H_24_O_2_	Fang Feng
JBD00198	Isoamylbenzene	C_11_H_16_	Dang Gui
JBD01030	Eugenol	C_10_H_12_O_2_	Sheng Jiang
JBD00350	(2S)-Flavanone	C_15_H_12_O_2_	Fang Feng
JBD00310	p-Cymen-8-ol	C_10_H_14_O	Fang Feng, Sheng Jiang
JBD00978	2-(4-hydroxyphenyl)ethyl 4-methoxybenzoate	C_16_H_16_O_4_	Qiang Huo
JBD00476	WLN: 4OVR	C_11_H_14_O_2_	Gan Cao, Sheng Jiang
JBD00233	L-Bornyl acetate	C_12_H_20_O_2_	Fang Feng, Qiang Huo, Sheng Jiang

### 3.5 The effect of the screened compounds on cell viability and inflammatory responses

**FIGURE 6:**
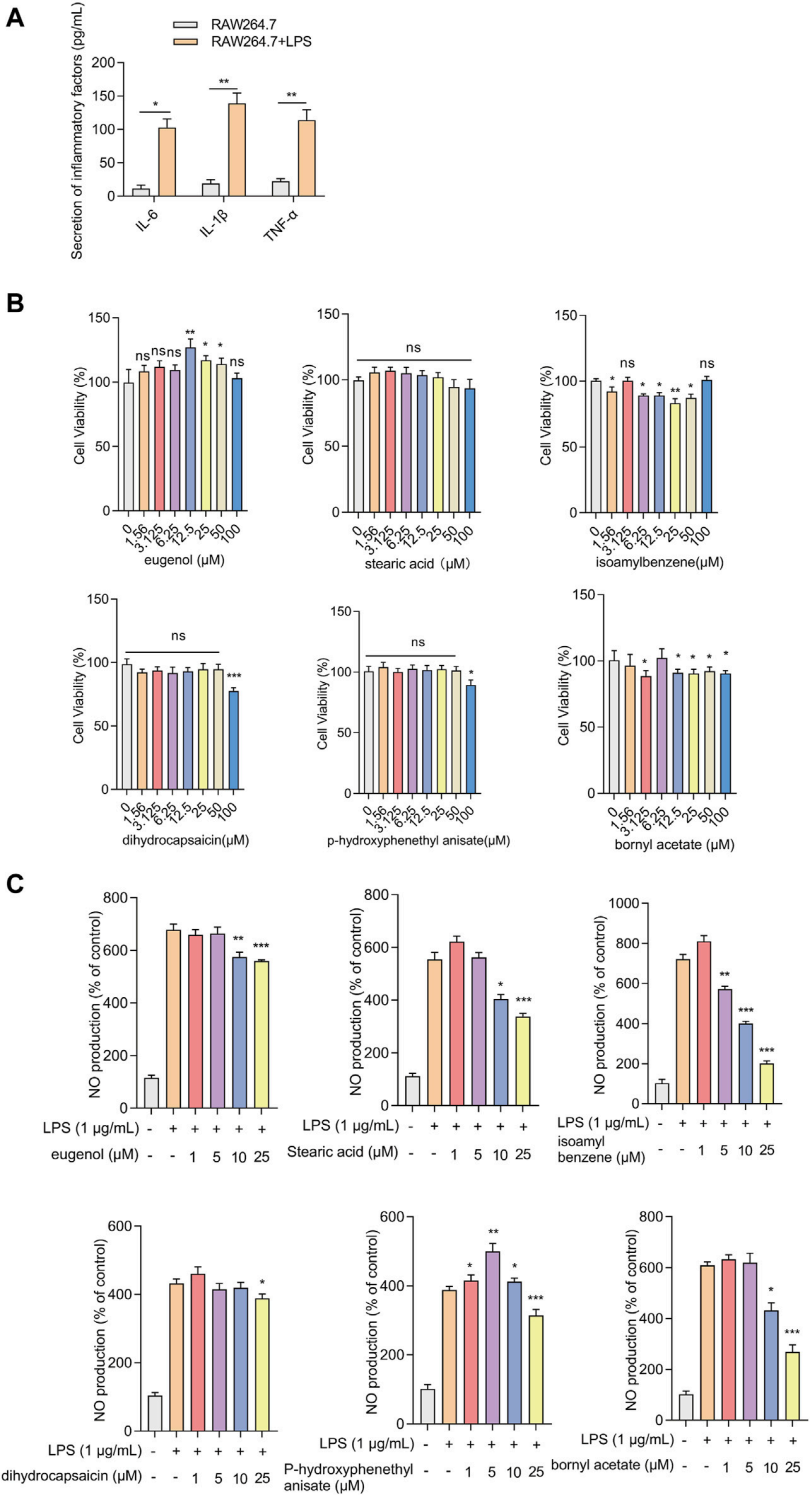
Effects of screened compounds on the inflammatory responses in LPS-induced RAW.264.7 cells **(A)** The IL-6, IL-1β, and TNF-α concentration in the cellular supernatant was examined by ELISA (n = 4). **(B)** The cells were cultivated with increasing concentrations of eugenol, stearic acid, isoamylbenzene, dihydrocapsaicin, p-hydroxyphenethyl anisate, and bornyl acetate (0, 1.56, 3.125, 6.25, 12.5, 25, 50 and 100 μM). Cell viability was measured by CCK-8 assay (n = 4). **(C)** NO production of RAW264.7 cells was measured after being treated with eugenol, stearic acid, isoamylbenzene, dihydrocapsaicin, p-hydroxyphenethyl anisate and bornyl acetate (1, 5, 10 and 25 μM, n = 4). The experiments were performed three times, and the error bars represent means ± SD. *p < 0.05, **p < 0.01, ***p < 0.001, ns: no significance.

The effects of the compounds on cell viability and inflammatory response were verified using LPS-induced RAW.264.7 cells. Because some compounds cannot be purchased or have no Chemical Abstracts Service (CAS) registry number, six selected compounds were utilized in the experiment: JBD01030 (eugenol), JBD00018 (stearic acid), JBD00198 (isoamylbenzene), JBD01230 (dihydrocapsaicin), JBD00978 (p-hydroxyphenethyl anisate), and JBD00233 (bornyl acetate). The secretion of IL-6, IL-1β, and TNF-α significantly increased in the LPS-induced RAW264.7 cells (Figure 6A). We then examined the cell viability of the RAW264.7 cells after treatment with JBD01030 (eugenol), JBD00018 (stearic acid), JBD00198 (isoamylbenzene), JBD01230 (dihydrocapsaicin), JBD00978 (p-hydroxyphenethyl anisate) and JBD00233 (bornyl acetate) (0, 1.56, 3.125, 6.25, 12.5, 25, 50 and 100 μM). There was no significant change in the cell viability of RAW264.7 cells after being treated with stearic acid, dihydrocapsaicin, or p-hydroxyphenethyl anisate (Figure 6B). Administration of isoamylbenzene and bornyl acetate significantly decreased the cell viability of RAW264.7 cells (Figure 6B). NO production significantly decreased after treatment with eugenol, stearic acid, and bornyl acetate at 10 and 25 μM in the LPS-induced RAW264.7 cells (Figure 6C). The administration of 3-methybutylbenzene at 5, 10, and 25 μM significantly decreased the NO production in LPS-induced RAW264.7 cells (Figure 6C). Moreover, treatment with dihydrocapsaicin and p-hydroxyphenethyl anisate at 25 μM decreased the NO production in LPS-induced RAW264.7 cells (Figure 6C). Intriguingly, administration of p-hydroxyphenethyl anisate at 1, 5 and 10 μM increased the NO production in LPS-induced RAW264.7 cells (Figure 6C). The above results showed that isoamylbenzene and bornyl acetate (BAC) both reduced cell viability and inhibited inflammation. Studies have shown that BAC has antioxidant and anti-inflammatory properties in different types of tissues and cells (Yang et al., 2018). Isoamylbenzene has only a benzene ring and a hydrophobic chain segment, so its water solubility is poor. It is an oily liquid, and it lacks other functional groups, such as hydroxyl, sulfhydryl, sulfonic acid and carboxyl. Oxygen in the ether bond can increase the water solubility of the drug, and aminos and amides can form hydrogen bonds with the target. In addition, the presence of benzene rings alone may present a risk of cancer. Therefore, we used BAC for the animal experiment verification.

### 3.6 Bornyl acetate alleviates joint damage in CIA mice

**FIGURE 7:**
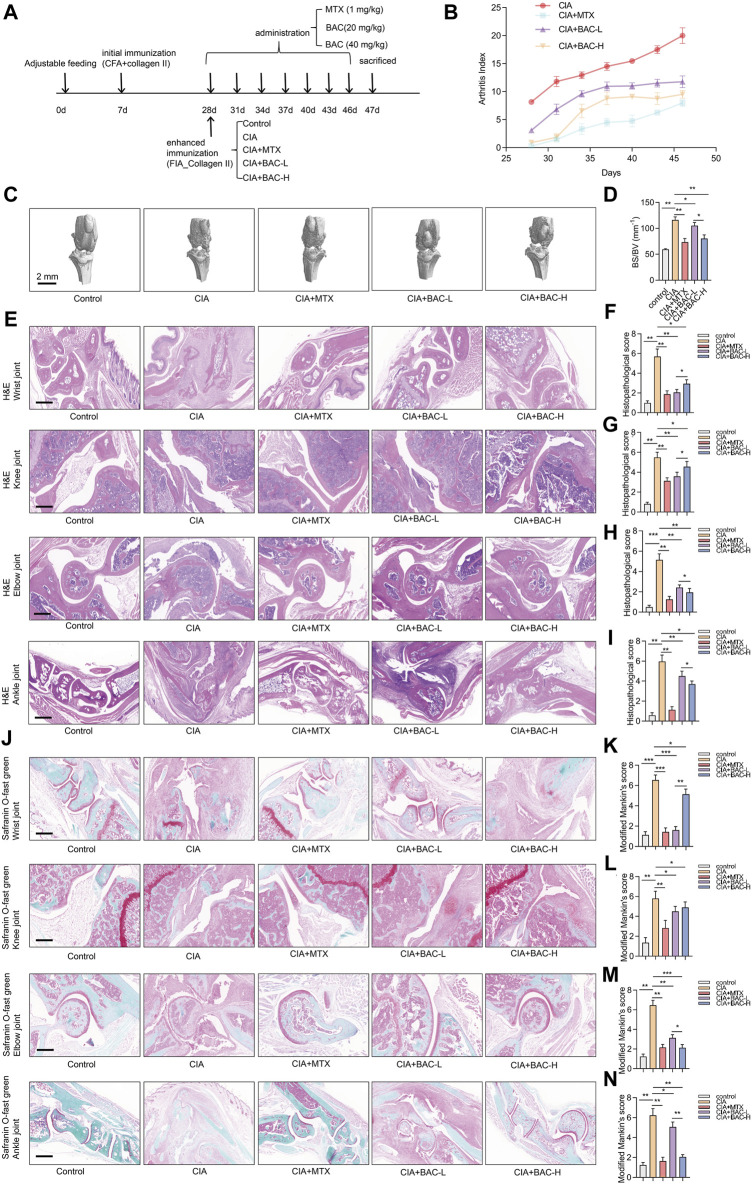
Bornyl acetate alleviates joint damage in CIA mice. **(A)** The timeline of constructing CIA mouse models, MTX treatment, and FZD treatment in this study (n = 6). **(B)** Arthritis index was examined after MTX and JBD treatment (n = 6). **(C)** Microcomputer tomography images of the knee joint of mice (n = 6). **(D)** Bone surface/volume ratio was calculated (BS/BV, mm-1) (n = 6). **(E)** H&E staining of wrist, knee, elbow, and ankle joint tissues in each group (n = 6). **(F–I)** Histopathological scores were measured independently by three pathologists blinded to the experiment (n = 6). **(J)** Wrist, knee, elbow, and ankle joint tissues were stained with safranin O-fast green (n = 6). **(K–N)** Modified Mankin’s scores of wrist, knee, elbow, and ankle joint tissues were measured (n = 6). The experiments were performed three times, and the error bars represent means ± SD. *p < 0.05, **p < 0.01, ***p < 0.001.

To verify the efficacy of BAC against RA, a CIA mouse model, which is widely used to clarify the mechanisms of RA and to explore the potential therapeutic targets, was established. The mice were then treated with MTX, the first-line treatment for RA, and two dosing regimens of BAC (Figure 7A).

The arthritis index (AI) of the CIA mice was higher than that of any other group. Furthermore, the AI of the MTX-treated CIA mice was lower than BAC-treated CIA mice. Importantly, the AI in the CIA + BAC-H group was lower than in the CIA + BAC-L group (Figure 7B). The effect of MTX or BAC on the knee joints of mice was measured by using micro-CT. The bone surface/volume (BS/BV) ratio of the CIA group was obviously higher than that of the control group. Administration of MTX or BAC significantly decreased the BS/BV ratio. Intriguingly, BAC at a high concentration was more effective in decreasing the BS/BV ratio (Figures 7C,D).

To further explore the effects of MTX and BAC on the CIA mice, the wrist, knee, elbow, and ankle joints of the mice were subjected to histological analysis by H&E and Safranin O staining. Compared with the control group, the joint structure was incomplete, the cartilage surface was rough, and a large number of inflammatory cells had infiltrated the joint tissues in the CIA group. Administration of MTX or BAC alleviated synovial hyperplasia and decreased the severity of cartilage damage and inflammatory cell infiltration (Figure 7E–N). Intriguingly, BAC at a low concentration was more effective in decreasing histopathological scores and modified Mankin’s scores of wrist and knee joints. BAC at a high concentration was more effective in decreasing histopathological scores and modified Mankin’s scores of the elbow and ankle joints of mice (Figure 7E–N).

### 3.7 BAC deactivated steroid hormone biosynthesis in CIA mice

**FIGURE 8:**
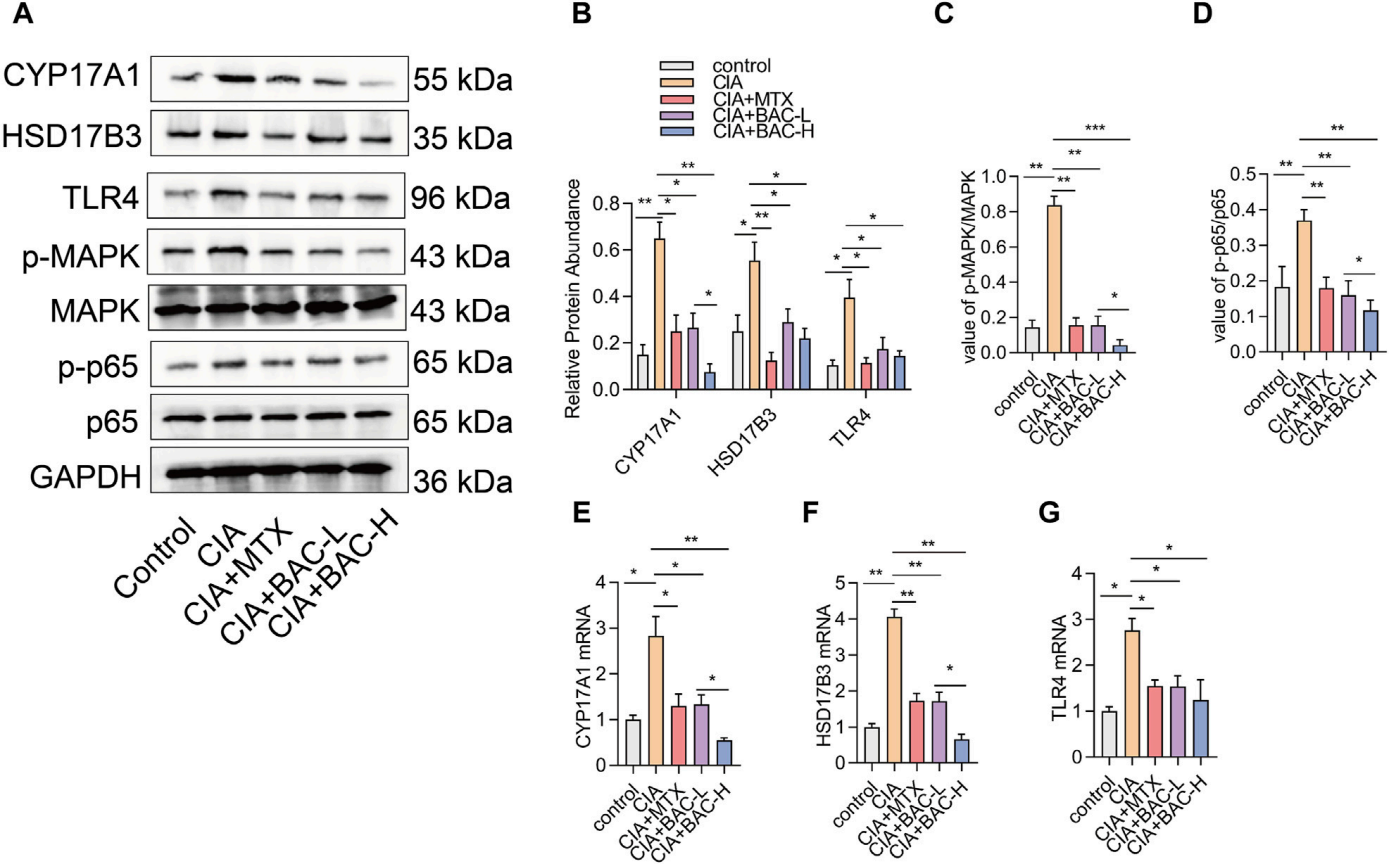
BAC deactivates steroid hormone biosynthesis in CIA mice. **(A–D)** Protein expression levels of CYP17A1, HSD17B3, TLR4, p-MAPK, MAPK, p-p65 and p65 were examined by Western blot (n = 6). **(E–G)** mRNA expression of CYP17A1, HSD17B3, TLR4, p-MAPK, MAPK, p-p65 and p65 was detected by RT-qPCR (n = 6). The experiments were performed three times, and the error bars represent means ± SD. *p < 0.05, **p < 0.01.

The bioinformatics analysis above revealed that the screened compounds exerted their most significant impact on the biosynthesis of steroid hormones. We selected several markers in steroid hormone biosynthesis to verify the effect of BAC on the levels of these proteins (including CYP17A1, HSD17B3, TLR4, MAPK, and p65). The expressions of CYP17A1, HSD17B3 and TLR4 increased in the CIA mice compared with the control mice. The phosphorylation of MAPK and p65 both significantly increased in the CIA mice compared with the control mice. However, administration of MTX and BAC decreased the expressions of these proteins (Figure 8). Importantly, BAC at a high concentration was more effective in decreasing the expressions of CYP17A1 and HSD17B3 and the phosphorylation levels of MAPK and p65 compared with BAC at a low concentration (Figure 8).

## 4 Discussion

In this study, we screened potentially active compounds in the JBD for the treatment of RA using systems pharmacology methods, and these compounds are primarily involved in multiple metabolic pathways, immune pathways, and signaling transduction pathways. Subsequently, experiments showed that isoamylbenzene and BAC significantly decreased cell viability and inflammation in RAW264.7 cells. Furthermore, we found that BAC alleviated joint damage in CIA mice and deactivated steroid hormone biosynthesis in CIA mice.

It has been reported that the JBD can treat with chronic lower back pain, external humeral epicondylitis, synovitis of the hip in children, ankylosing spondylitis, gout, and other bone diseases. In addition, it decreases inflammatory cytokines, such as TNF-α and IL-1, in the serum of rats induced by Freund’s adjuvant (Xiao and Ye, 2021). Then Zini Juan-Bi Tongluo decoction combined with MTX and salazine sulfopyridine relieves the main clinical symptoms of early RA with damp-heat arthralgia and reduces the level of adipokines in the serum (Xiao and Ye, 2021). The CIA mouse model is the most commonly studied autoimmune model of RA because it shares several pathological features with RA, including synovial hyperplasia, cartilage degradation, mononuclear cell infiltration, and bone destruction (Qin et al., 2022). In this study, we successfully established the CIA mouse model. We demonstrated that BAC or MTX treatment effectively inhibited joint destruction and inflammatory cell infiltration in CIA mice, indicating that BAC alleviates the symptoms of RA.

The enrichment analysis results indicated that steroid hormone biosynthesis, linoleic acid metabolism, and arachidonic acid metabolism are the three primary pathways that these compounds are mainly involved in. Steroid hormones, especially estrogen, play crucial roles in the pathogenesis of RA (Sparks, 2019), which primarily affects skeletal performance and immune functions (Islander et al., 2011). Single nucleotide polymorphisms associated with steroid hormones are connected to the formation of bone erosions in RA and contribute to predicting the progression of the disease (Sanchez-Maldonado et al., 2019). Linoleic acid is an unsaturated fatty acid. The association between linoleic acid and inflammation remains controversial. Some researchers have proposed that linoleic acid possesses anti-inflammatory properties, as demonstrated by reductions in IL-1 and IL-6 (Rodrigues et al., 2012). In addition, conjugated linoleic acid, an isomer of linoleic acid, has been found to decrease TNF-α levels in RA patients, exerting an anti-inflammatory effect in active RA (Islander et al., 2011). However, it is essential to note that linoleic acid also serves as a precursor to pro-inflammatory compounds such as arachidonic acid and prostaglandin E2, potentially exacerbating inflammation (Jandacek, 2017). Arachidonic acid (AA) is a polyunsaturated fatty acid that plays a significant role in the inflammatory process, as it is a precursor to pro-inflammatory eicosanoids, such as prostaglandins and leukotrienes (Wang et al., 2021). These eicosanoids are involved in the regulation of immune responses and inflammation (Ye et al., 2021). Studies have shown that increased AA levels and the subsequent production of pro-inflammatory eicosanoids contribute to the chronic inflammation observed in RA joints (Lei et al., 2023).

Next, we conducted *in vitro* experiments to explore the effects of the screened compounds on inflammation in LPS-induced RAW264.7 cells. The secretion of IL-6, IL-1β, and TNF-α, which play important roles in the pathogenesis and development of RA, significantly increased in LPS-induced RAW264.7 cells. The greatest challenge in revealing the effective material basis for TCM is the extremely complicated compositions. Only a few compounds that have favorable physicochemical properties and sufficient content in TCM can be absorbed by the body and transported to the target organs by the plasma to exert the related activities, although they contain multiple ingredients. We screened the active compounds of the JBD, like eugenol, stearic acid, isoamylbenzene, dihydrocapsaicin, p-hydroxyphenethyl anisate, and BAC. The cell viabilities of RAW264.7 cells were then examined after being treated with these active compounds. BAC administration and isoamylbenzene significantly decreased cell viability.

NO has been considered an endogenous gaseous bio-signaling molecule and exhibits concentration-dependent biological effects. Induction of iNOS in the synovial lining layer, infiltrated cells, and cartilage by inflammatory cytokines (IL-1β, IL-6, TNF-α) releases large amounts of NO (Teng et al., 2011; Arroul-Lammali et al., 2017). It has been demonstrated that there is an association of the increased serum nitrite (NO surrogate) in patients suffering from active RA with disease activity. Over-production of NO can cause negative effects, such as cell apoptosis, chronic inflammatory disorders, and tissue destruction, which are closely linked to RA. As a consequence, selective clearance of the overproduction of NO may act as a novel therapeutic strategy for RA therapy (Arroul-Lammali et al., 2017). NO production increased after LPS was induced, an indicator of inflammation. NO production could be inhibited by the administration of active compounds that have anti-inflammatory effects. Our results demonstrated that eugenol, stearic acid, isoamylbenzene, dihydrocapsaicin, p-hydroxyphenethyl and BAC decreased NO production in LPS-induced RAW264.7 cells.

BAC is the primary volatile constituent in numerous conifer oils and several traditional Chinese herbs, such as Fructus Amomi and Houttuynia Cordata (Zhao et al., 2023). BAC has a variety of pharmacological activities, acting as an anti-inflammatory, anti-microbial, and neurotransmitter modulator. Several studies have demonstrated its ability to attenuate inflammation by inhibiting pro-inflammatory mediators and cytokines, such as IL-1β, IL-6, IL-11, and TNF-α (Chen et al., 2014; Yang et al., 2014; Zhao et al., 2023). Treatment with BAC protects against IL-1β-induced inflammation in chondrocytes by elevating the expression of IL-11 through activating AP-1 transcriptional activity, indicating the therapeutic potential of BAC in patients with osteoarthritis (Yang et al., 2014). Pretreatment with BAC has been shown to significantly improve interstitial edema, aveolar hemorrhage, and inflammatory cell infiltration in the lungs of LPS-induced acute lung injury (ALI) mice (Chen et al., 2014). In addition, BAC has been shown to suppress the expression of inducible nitric oxide synthase and cyclooxygenase-2, enzymes involved in the production of inflammatory mediators like nitric oxide and prostaglandins, respectively (Zhao et al., 2023). Moreover, BAC exhibits notable antimicrobial effects against a wide range of pathogens, including bacteria, fungi, and some viruses (C Elbouzidi et al., 2024; Benali et al., 2023). Studies have highlighted its ability to disrupt bacterial cell membranes, inhibit biofilm formation, and interfere with viral replication processes (C Elbouzidi et al., 2024). These antimicrobial properties suggest its potential utility in the treatment of various infectious diseases, either as a standalone therapy or in combination with conventional antibiotics or antiviral agents. Furthermore, BAC has shown promise in modulating neurotransmitter activity and exerting sedative effects, and this could be beneficial in managing conditions such as anxiety, insomnia, and stress-related disorders (Granger et al., 2005; Zhang et al., 2021b). Its ability to interact with gamma-aminobutyric acid (GABA) receptors and serotonin receptors may contribute to its anxiolytic and hypnotic properties (Granger et al., 2005).

Among the 20 pathways predicted by the bioinformatics analysis, steroid hormone biosynthesis had the most significant change. We selected several markers in steroid hormone biosynthesis to verify the effect of BAC on the levels of these proteins, including CYP17A1, HSD17B3, TLR4, MAPK, and p65. In this study, our results demonstrated that BAC decreased the expression levels of CYP17A1, HSD17B3, and TLR4, as well as the phosphorylation of MAPK and p65. Intriguingly, the phosphorylation of MAPK, p65, and the expression level of CYP17A1 were significantly lower in a high concentration of JBD compared with a low concentration of JBD. A recent study suggested that Lamiophlomis rotata may exhibit anti-inflammatory properties in RA through sphingolipid and steroid hormone regulation, highlighting a potential new research direction for RA (Zhou et al., 2023). It has been demonstrated that RA has decreased androgen levels. The decisive step governing androgen synthesis is the 17,20-lyase activity of the CYP17A1 gene-encoded enzyme cytochrome P450 17A1 (Stark et al., 2015). It has been demonstrated that hyperactivation of TLR4 triggers the production of various inflammatory factors that are associated with the development of a variety of diseases, such as rheumatoid arthritis and cardiovascular diseases (Zhang et al., 2022). Moreover, BAC inhibits the NF-κB signaling pathway by affecting the phosphorylation of IKB, inhibits the MAPK signaling pathway through suppressing the phosphorylation of ERK1/2, JNK, and p38 induced by LPS, downregulates pro-inflammatory cytokines such as TNF-α, IL-1β and IL-6, and reduces NO production (Wang et al., 2020).

Regarding its toxic profile, BAC is generally considered safe when used in appropriate doses and formulations (Zhao et al., 2023). There is no direct report on the toxicity of BAC in humans and only one report on the acute toxicity of BAC in mice (Wu et al., 2004). This study showed that BAC has a potential therapeutic effect on RA, and future clinical research needs to clarify its specific clinical efficacy, side effects, toxicity, and other information. In addition to BAC, other compounds screened in this study also have potential effects on the treatment of RA. Future studies need to verify the efficacy of these compounds and explore their underlying mechanisms. There are some limitations in this study. First, the chemical components in the JBD we collected may not have been sufficient. Second, the accuracy of the compound-target prediction needs to be improved. Finally, we did not model the metabolic process of the screened compounds. We believe that using network pharmacology approaches to identify potentially active compounds combined with experimental verification can speed up the process of screening natural active ingredients. Our future work will optimize the process of virtual screening of compounds and use metabolic modeling methods to explore the effects of compounds on metabolic processes.

## 5 Conclusion

In summary, in this study, we screened multiple potentially effective compounds of JBD for the treatment of RA using an *in silico* analysis. Cellular experiments proved that isoamylbenzene and bornyl acetate significantly decreased cell viability and inhibited inflammation. Further experiments showed that BAC decreased steroid hormone biosynthesis in CIA mice. This study provides a methodological reference for the screening of active compounds in TCMs. The method proposed in this study can be used to screen the active compounds of other prescriptions.

## Data Availability

The original contributions presented in the study are included in the article/Supplementary Material, further inquiries can be directed to the corresponding authors.

## Supplementary material

SUPPLEMENTARY TABLE S1 All compounds in JBD and their molecular physicochemical and ADMET properties.

SUPPLEMENTARY TABLE S2 Preliminary screened compounds in JBD and their molecular physicochemical and ADMET properties.

SUPPLEMENTARY TABLE S3 Target prediction results of filtered compounds.

SUPPLEMENTARY TABLE S4 Detailed information on the rheumatoid arthritis pathogenic genes.

SUPPLEMENTARY TABLE S5 Enriched KEGG pathways of hub target genes.

SUPPLEMENTARY TABLE S6 Enriched GO biological processes of hub target genes.

SUPPLEMENTARY TABLE S7 Cumulative intensity of the filtered compounds on rheumatoid arthritis pathogenic genes.

SUPPLEMENTARY TABLE S8 Cumulative interaction intensity flow on metabolic pathways.
